# Advances in multiscale image processing and its effects on image quality in skeletal radiography

**DOI:** 10.1038/s41598-022-08699-8

**Published:** 2022-03-18

**Authors:** Susan Notohamiprodjo, K. M. Roeper, F. G. Mueck, D. Maxien, F. Wanninger, B. Hoberg, L. Verstreepen, K. M. Treitl, F. Fischer, O. Peschel, S. Wirth

**Affiliations:** 1 Department of Nuclear Medicine, Klinikum rechts der Isar, Technical University of Munich, Ismaninger Str. 22, 81675 Munich, Germany; 2 Department of Ophthalmology, Klinikum rechts der Isar, Technical University of Munich, Munich, Germany; 3 Department of Radiology, HELIOS Klinikum München West, Munich, Germany; 4 Radiologisches Zentrum München, Munich, Germany; 5 Agfa-Gevaert HealthCare GmbH, Munich, Germany; 6 Agfa HealthCare Germany GmbH, Bonn, Germany; 7 Agfa NV, Mortsel, Belgium; 8 Department of Radiology, University Hospital of Munich, LMU Munich, Munich, Germany; 9 Institute of Forensic Medicine, LMU Munich, Munich, Germany; 10 Institute of Radiology, Schwarzwald-Baar Klinikum, Villingen-Schwenningen, Germany

**Keywords:** Bone imaging, Radiography

## Abstract

Multi-frequency processing (MFP) leads to enhanced image quality (IQ) of radiographs. This study is to determine the effect of third generation MFP (M3) on IQ in comparison to standard second-generation MFP (M2). 20 cadavers were examined and post-processing of radiographs was performed with both M2 and M3. Three readers blinded to the MFP used for each image independently compared corresponding image pairs according to overall IQ and depiction of bony structures and soft tissue (+ 2: notably better > 0: equal > − 2: notably worse). A significant deviation of the median grade from grade 0 (equal) (p < 0.01) for each evaluator A, B and C speaks against an equal image quality of M2- and M3-images. M3-images were categorized with better grades (+ 1, + 2) in 87.7% for overall image quality, in 90.4% for soft tissue and 81.8% for bony structures. M3 images showed significant higher averaged SNR and CNR for all investigated lower extremities than that of M2 images (0.031 < p < 0.049). The newest generation of MFP leads to significantly better depiction of anatomical structures and overall image quality than in images processed with the preceding generation of MFP. This provides increased diagnostic accuracy and further decreased radiation exposure.

## Introduction

Despite the era of Magnetic Resonance and Computed Tomography in medical imaging, conventional radiography still plays an essential role in dental, chest and skeletal diagnostics. In several countries, radiography is the most frequently performed medical X-ray examination^1–3^. The digitalization of radiography brought advantages such as teleradiology, electronic archiving and the ability to adjust brightness and contrast of the image on the screen^4^. However, the transition from screen-film to digital radiography provided several challenges. The wider dynamic range of digital radiography than in screen-film decreased the vulnerability of high-contrast structures to exposure dose. On the one hand diagnostic image quality is preserved in a wider exposure dose range. On the other hand this led to a phenomenon known as “dose-creep”, the tendency to overexposure in digital radiography^5–7^. Furthermore the wider dynamic range of digital radiography led to a better differentiation of structures with highly varying absorption levels^8,9^. However, because of this high dynamic range the image appears to lack contrast. In comparison to screen-film the spatial resolution of digital radiography is limited by the pixel size^8^. To overcome the disadvantages of digital radiography compared with screen-film further development of plate detector technology and digital post-processing of the raw data is mandatory.

Advances in detector technology and digital post-processing led to enhanced image quality. This allowed an increase of diagnostic accuracy and a decrease of radiation exposure according the as low as reasonably achievable (ALARA) principle^10,11^. Next to Unsharp Mask Filtering, Multi-frequency processing (MFP) is a commonly used method of digital post-processing using Laplace segmentation of the image data^11–14^. Several manufacturers developed a MFP algorithm adjusted to their digital direct radiography (DDR) systems, pioneered by AGFA Healthcare (Multiscale Image Contrast Amplification (MUSICA))^15^, Philips (Unified Image Quality Enhancement (UNIQUE))^16^ and Fujifilm (Multi-Objective Frequency Processing (MFP))^17^. MFP allows the reduction of strong contrasts and image noise and the enhancement of edges and subtle contrasts. It leads to increased overall image quality, to a more homogenous visual impression of the whole radiograph from center to the edges, to a more detailed depiction of fine structures and to less artifacts caused by superposition of structures, improper patient positioning or metallic objects^18,19^.

The first generation of MFP required manual adjustments of parameters including contrast, brightness, edge sharpness and noise reduction. To overcome the dependence of the image appearance from the user’s experience, the next generation of MFP automatically adjusts the parameters after analyzing the chosen exposure setting and the characteristics of the raw data without requiring further information from the user regarding chosen exposure settings. This led to highly reproducible image quality less vulnerable to the radiographic projection, patient positioning and the presence of contrast media or metallic objects. As digital technology advances, optimized algorithms of the most recent generation of MFP have been developed. Those improvements are expected to simplify and accelerate the clinical workflow, to maintain a constantly high level of image quality and to improve diagnostic certainty.

This clinical experimental study is to compare the performance of a new generation of MFP to the recently established MFP generation in direct radiographic skeletal imaging at the example of MUSICA 2 and MUSICA 3 (AGFA Healthcare, Mortsel, Belgium—The indices 2 and 3 of the MUSICA represent the generation of the MFP).

## Results

Average DAP (cGy*cm^2^) was 122.1 in pelvis, 9.6 in knee and 2.9 in ankles.

ICC for overall image quality, joint space, bony protrusion, fat-muscle layer were 0.81, 0.81, 0.76 and 0.86.

The third generation MFP (M3-radiographs) was evaluated with superior scores (+ 1, + 2) in 87.7%, as equal (0) in 10.1% and with inferior scores (− 1, − 2) in 2.2%. Vice versa the second generation MFP (M2-radiographs) was evaluated with superior scores (+ 1, + 2) in 6.4%, as equal (0) in 10.3% and with inferior scores (− 1, − 2) in 83.3%. The differences between the constellations M2 and M3-radiograph on the left screen in regard to the distribution of grades was not significant (p = 0.14).

**Figure 1:**
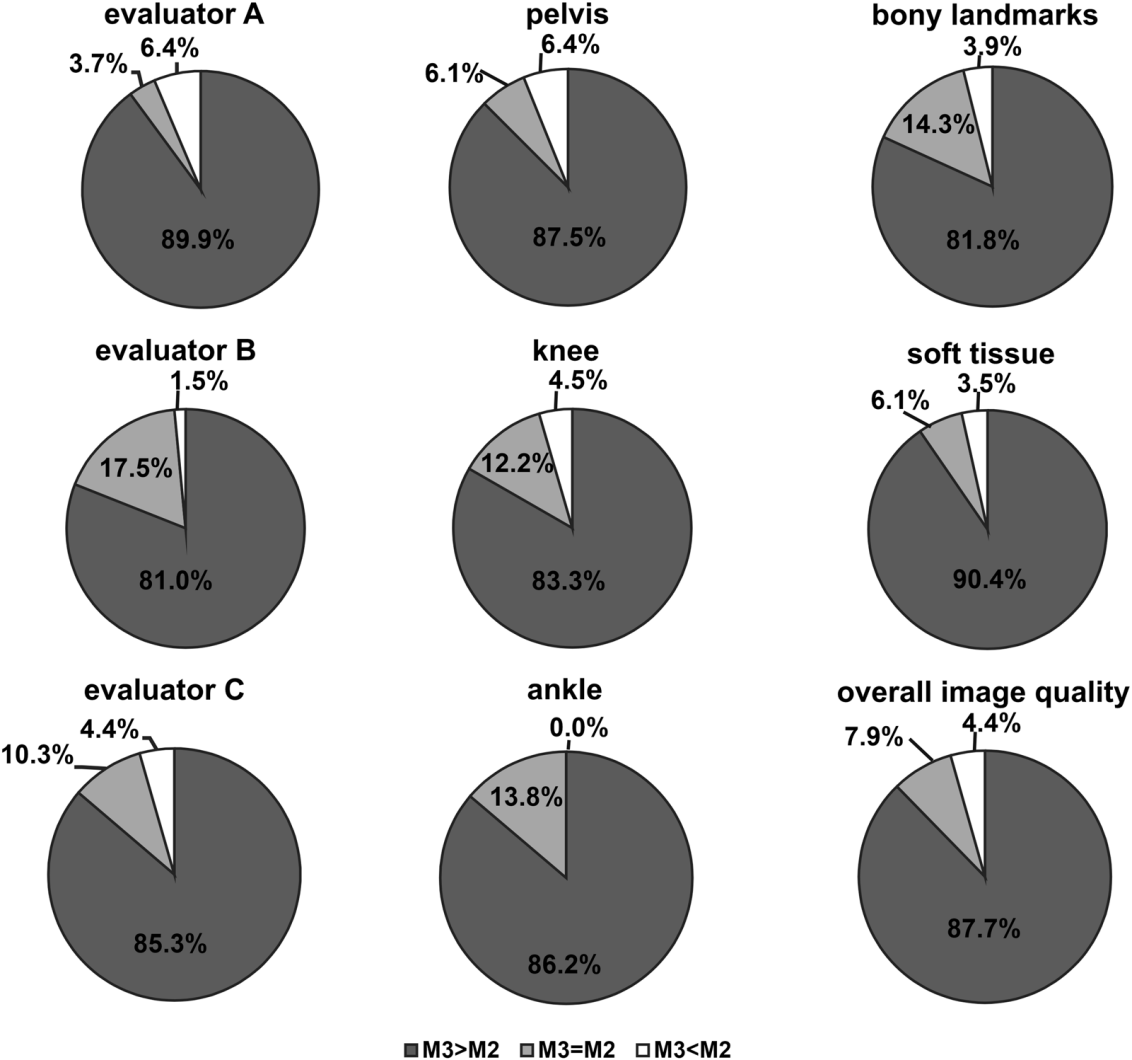
Distribution of grades. The distribution of grades itemized on evaluators, anatomical region, bony landmarks, soft tissue and overall image quality. In a majority (> 80%) M3-processed images received superior grades (+ 1, + 2) than M2-processes images.

All grades (− 2, − 1, 0, + 1, + 2) given for M3-radiographs were 0.4%, 5.9%, 3.8%, 55.5% and 34.4% by evaluator A, respectively 0%, 1.6%, 17.5%, 67.5% and 13.4% by evaluator B and 0.3%, 4.1%, 9.2%, 65.5% and 20.9% by evaluator C and 0.2%, 3.9%, 10.1%, 62.9% and 22.9% for all evaluators. The distribution of grades for each body region, anatomical landmarks and overall image quality show a majority of higher grade given to M3-radiographs (> 80%) (Fig. 1). The average and median scores given by the evaluators, for the body region, overall image quality and anatomical landmarks are shown in Table 1. The median grade given by the evaluators differed significantly from grade 0 (p < 0.05).

**Table 1:** Descriptive statistics of given scores. The average and median of the scores given by the evaluators, for the body region, overall image quality and anatomical landmarks were calculated. They differed significantly from grade 0 (p < 0.05) in all categories.

	Average ± standard deviation	Median	25th percentile	75th percentile
Evaluator A	1.18 ± 0.79	1	1	1
Evaluator B	0.93 ± 0.60	1	1	1
Evaluator C	1.04 ± 0.62	1	1	1
All evaluators	1.05 ± 0.72	1	1	1
Pelvis	1.09 ± 0.76	1	1	2
Knee	1.00 ± 0.74	1	1	1
Ankles	1.09 ± 0.60	1	1	1
Bones	1.00 ± 0.73	1	1	1
Soft tissue	1.18 ± 0.71	1	1	2
Image quality	1.04 ± 0.68	1	1	1

**Figure 2:**
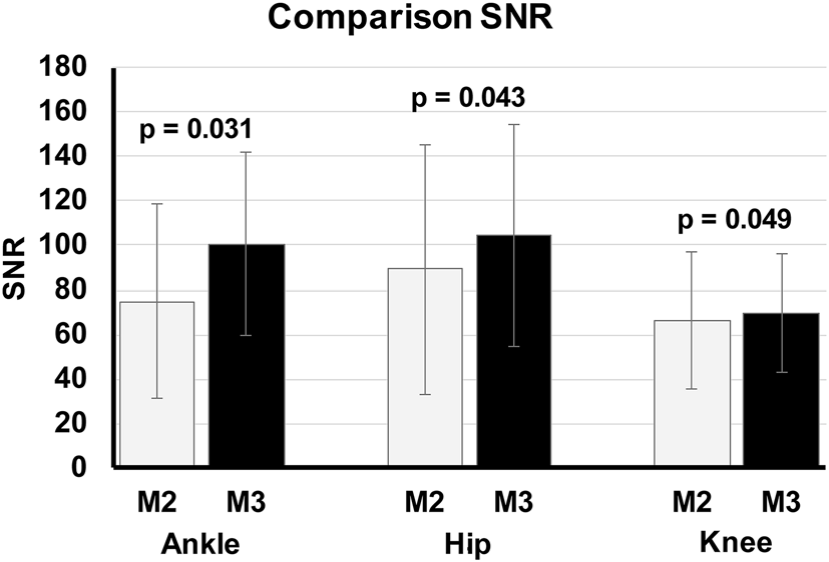
Comparison SNR between MUSICA 2 and MUSICA 3 images. Average SNR of M3 images was slightly higher than SNR of M2 images. Statistical analysis showed still significant differences.

**Figure 3:**
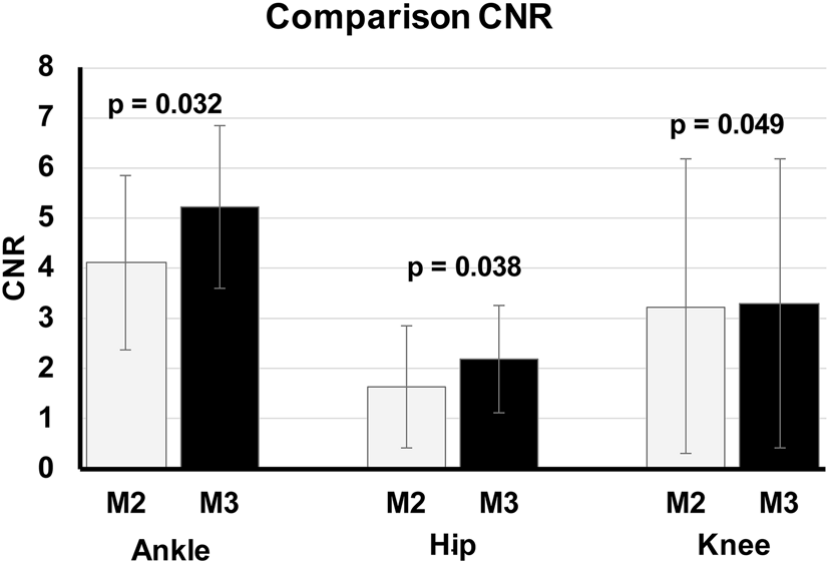
Comparison CNR between MUSICA 2 and MUSICA 3 images. Average CNR of M3 images was slightly higher than CNR of M2 images. Statistical analysis showed still significant differences.

The SNR and CNR of the M3 images were all slightly higher than that of M2 images. The comparison of the SNR of the image duplets (paired M2 and M3 image) showed however still significant differences in SNR with p = 0.043 for hip image duplets, p = 0.049 for knee image duplets and p = 0.031 for ankle image duplets (Fig. 2). Similar results were obtained for CNR comparison of the image duplets with still significant differences in CNR with p = 0.038 for hip image duplets, p = 0.049 for knee image duplets and p = 0.032 for ankle image duplets (Fig. 3).

**Figure 4:**
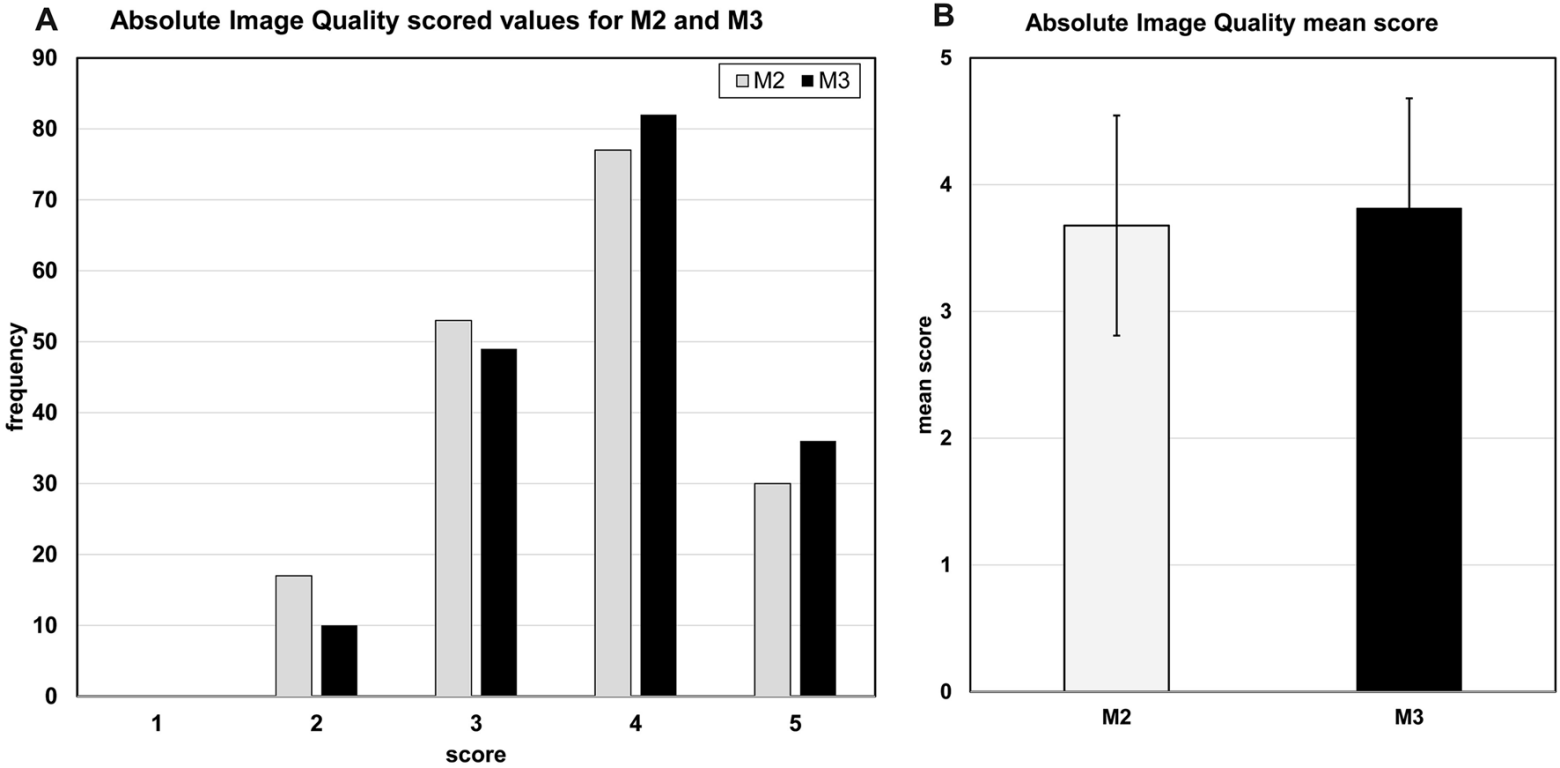
Visual absolute grading of image quality for MUSICA 2 and MUSICA 3 images. (**A**) More M3 images were graded with scores 4 and 5 (high quality) and less M3 images were graded with scores 2 and 3 (lower quality) compared to M2. No images of M2 and M3 were graded as non-diagnostic. (**B**) The observers tend to have slightly more preference for M3 than for M2 images.

The visual absolute image quality score independently for M2 and M3 showed more M3 images were graded with scores 4 and 5 (high quality). Less M3 images were graded with scores 2 and 3 (lower quality) compared to M2. No images of M2 and M3 were graded as non-diagnostic (Fig. 4A). The comparison of the mean score showed that the evaluators tend to have slightly more preference for M3 than for M2 (Fig. 4B).

## Discussion

This study’s aim to compare the performance of a new generation of MFP to the recently established MFP generation in skeletal radiography significantly showed the superiority of MUSICA 3 compared to MUSICA 2. Image quality analyses in a post-mortem-study at different exposure levels was published previously^20^. The image quality was resilient against tube voltage variations. To compare the performance of the postprocessing software M2 and M3, it is sufficient to use one exposure setting of the exposed raw image data and postprocessed them consecutively with M2 and M3 algorithm.

The quantitative assessment of the image quality in terms of SNR and CNR demonstrated significant higher values of M3 than M2 images (Figs. 2 and 3).

The visual grading of the images assigned by 3 radiologists revealed that more than 80% of superior grades (+ 1, + 2) were given to M3-processed radiographs with high significance (p < 0.05). Superior grades for M3-radiographs were given in 81.8% for the delineation of bony landmarks, 87.7% for overall image quality and especially in the soft tissue contrast with 90.4%. The agreement between the evaluators was high (ICC > 0.76) in all aspects (overall image quality, bony landmarks, soft tissue).

The Wilcoxon test showed no significant difference in the given grades regarding the monitor used and the order of display. This excludes a bias caused by these features and hints to the great impact of the image quality difference on the distribution of grades.

All results of this qualitative and quantitative assessment showed that the performance of MUSICA 3 is superior to MUSICA 2. This in turn demonstrates the importance of the algorithm implemented in the MFP to post-process radiographs. An improved MFP-algorithm contributes to better image quality without changing exposure parameters and exposure dose. This quantitative improvement of image quality by MUSICA 3 was shown in this study to have a significant impact on the visual impression of the radiographs.

Sensakovic et al.^18^ found MUSICA 2 to yield higher image quality than with Optima XR220amx (GE Healthcare, Waukesha, WI), especially in radiographs obtained with low exposure dose. In 65% of the radiographs with undiagnostic image quality, MUSICA 2 was able to improve the image quality to a more adequate level^18^. Phantom studies confirmed the potential of MFP for further dose reduction in digital radiography up to 61%^21,22^. The impact of the next generation of MFP on diagnostic accuracy and potential for further dose reduction needs to be further investigated.

**Figure 5:**
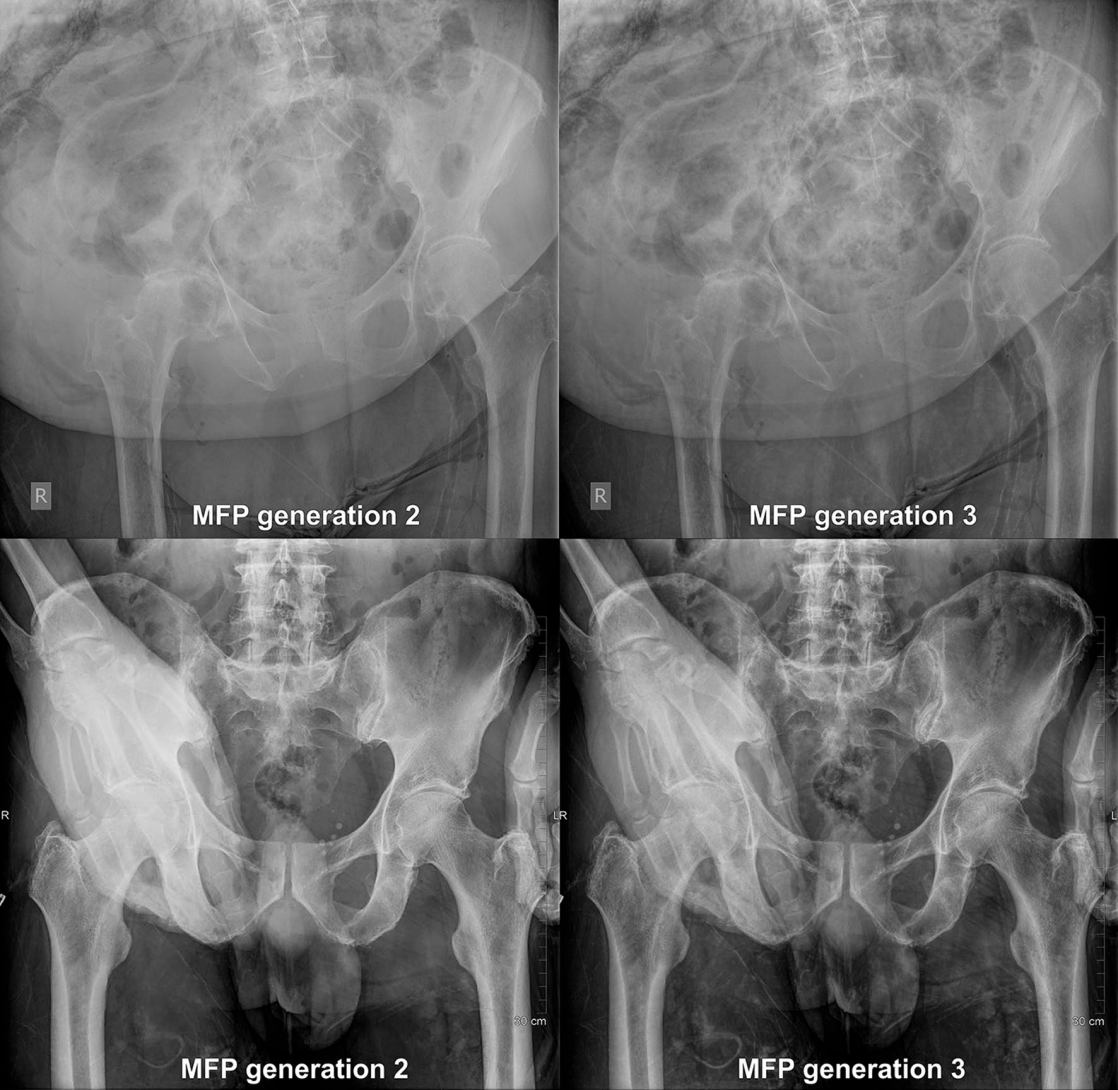
Exemplary image pair of an improper positioned, obese body and superimposed structures. The left images were post-processed with MUSICA 2. The right images were post-processed with MUSICA 3. Note MUSICA 3 depicts more details in obese body and in region with superimposed structures.

An explanation why the evaluators preferred MUSICA 3 over MUSICA 2 reformatted images may be because of a better performance especially in adipose and improper positioned patients and in superimposed structures (Fig. 5). In M3-processed images soft tissue appears more translucent than in M2-processed images^11^. Thus, the thickness of the soft tissue seems to have less impact on the delineation of the bone structures, especially in the pelvic region with superposition of the bones by inner organs. The soft tissue mantle seems to have a more homogenous appearance and improved contrast between fat and muscle.

However, the mechanisms responsible for the image quality improvement are not known, as details of the algorithm are mostly kept secret. For MUSICA 3 the manufacturer writes in an information brochure that they have not changed the mathematical principle of the multiscale contrast amplification, but they used a new mathematical algorithm with the fractional multiscale processing and provide a wider dynamic range^23^. However, a simple widening of the dynamic range leads to a decrease of image contrast and cannot be the sole reason for this change of visual appearance of the radiographs. The improved depiction of bones and more translucent impression of the soft tissue mantle hints to multiple changes within the dynamic range. For example, a stronger contrast between the gray value ranges of bones and soft tissue may result in brighter bones and in darker soft tissue. A slightly increased contrast between the gray value ranges of cortical and trabecular bone may result in an improved delineation of those structures. A decreased contrast between the gray value ranges of several soft tissues may result in an increase of homogeneity of the soft tissues. A more detailed processing of a great range of particular dynamic bands may be the main reason for the effects of MUSICA 3 on the visual impression of the images.

## Limitations

However, the transferability of this study’s results to other next generation MFP algorithms is limited, because the newest versions of MFP algorithms of other vendors were not available for this study. Examples of other MFP algorithms are Helix Advanced Image Processing (GE Healthcare), Unified Image Quality Enhancement (UNIQUE) (Philips), EVP Plus (Carestream), Multi-Objective Frequency Processing (MFP) (Fuji) and DiamondView MAX (Siemens Healthineers). Further comparative studies including diverse MFP algorithms are encouraged to assess, if the recent revisions of the MFP algorithms are a true technologic advancement or just modifications of the status quo.

## Conclusion

In this clinical experimental study on skeletal digital radiography, radiologists independently preferred images post-processed with the successor version of MUSICA, a multi-frequency processing tool, in regard of overall image quality and the depiction of anatomical landmarks, especially in adipose and improper positioned patients. A greater dynamic range and modifications in the fractional multiscale processing may explain this better performance. To assess the impact on diagnostic accuracy and the transferability to other vendors MFP algorithms, further investigations are encouraged.

## Materials and methods

### Study population

This study’s population was published in a previous study^20^. This post-mortem study was approved by the institutional ethical review board (Ethikkommission bei der Medizinischen Fakultät der LMU München, chairmen: Prof. Dr. W. Eisenmenger, Prof. Dr. R. M. Huber, Prof. Dr. C. Wendtner). Informed written consent of next of kin of deceased patients was waived by the institutional ethical review board (Ethikkommission bei der Medizinischen Fakultät der LMU München, chairmen: Prof. Dr. W. Eisenmenger, Prof. Dr. R. M. Huber, Prof. Dr. C. Wendtner). All methods were performed in accordance with the relevant guidelines and regulations. Virtual and conventional autopsy were ordered by the public prosecutor’s office. All cadavers referred by the Institute for Forensic Medicine of the Ludwig-Maximilians-University Munich from August till September 2018 were initially included. Exclusion criteria were destruction of the legs, infantile bodies, weight more than 100 kg, supine position not possible, advanced decay, affection of the clinical routine. All cadavers finally included to the previous study (n = 20, with 20 pelvis, 13 right knee, 12 left knee, 14 ankle images) were included to this study. Population data provided by the forensic medicine department such as age, weight, height, elapsed time, location, circumstances and cause of death have been published previously^20^.

### Examination procedure

Post-mortem radiography is a challenging task for the staff involved in the examination procedure. Because of the strict schedule determined by the prosecutor’s office and the daily workflow in the forensic medicine department, all post-mortem X-ray examinations had to be performed during the clinical routine in the radiology department.

All radiographs were performed with DX-D600 (AGFA HealthCare, Mortsel, Belgium), a direct digital radiography (DDR) system with fixed flat-panel detector Varian CsI (pixel size 139 µm) (AGFA HealthCare, Mortsel, Belgium). The Bucky table was covered with a plastic sheet to preserve hygienic conditions. The cadavers were transported in an opaque body bag within a coffin by the funeral directors. The procedure was hidden from living patients and other bystanders as well as possible. The body was moved from the coffin to the Bucky table in supine position by the funeral directors. Anatomical landmarks had to be identified via palpation to estimate the correct collimation. Anatomical landmarks were the iliac crest and greater trochanter for pelvis, patella for knee and mortise and hell for ankle. The zipper of the body bag was shifted to an appropriate spot to avoid superposition with the bones in the radiographs.

The examination parameters for pelvis, single knee and both ankles complying with national regulations in radiation protection^24^ are subsumed in Table 2. All images were post-processed with both Multiscale image contrast amplification (MUSICA) Version 2 and Version 3 (AGFA HealthCare, Mortsel, Belgium) with default settings (Contrast = 0, Brightness = 0, Sharpness = 0) resulting in 59 duplets of images in a total number of 118 radiographs. Dose parameters were recorded in dose-area-product (DAP, [cGy*cm^2^]). All patient data were anonymized.

**Table 2:** Examination protocols. Complying with the national regulations in radiation protection, pelvis/knee and ankles were examined according the recommendations for big joints and for small joints, respectively.

	Pelvis (a.p.)	Knee (a.p.)	Ankles (a.p.)
Tube voltage (kV)	80	80	60
Exposure mode	Automatic exposure control	Automatic exposure control	Manual exposure (1.6 mAs)
Anti-scatter grid	52 lines/cm, ratio 8:1, aluminum, f_0_ = 100 cm	52 lines/cm, ratio 8:1, aluminum, f_0_ = 100 cm	None
SID	1.15 m	1.15 m	1.15 m
Ionization chamber	Left and right	Middle	None

After the examination the room was aired, the funeral directors moved the body back to the coffin and transferred the body to the dissecting rooms of the forensic medicine department.

If this whole procedure is not possible or interrupted because of a simultaneous medical emergency or other affection of the clinical routine, the examination was cancelled.

### Multiscale image contrast amplification

MUSICA is a tool for further digital image processing^15^. At first the image is analyzed via histograms, assessment of noise and contrast-to-noise ratio, mask calculations and enhancement factors^25^. After enhancement of the raw image, a Laplace pyramid segmentations results in multiple frequency bands^26^. Each frequency band can be further processed independently. The process includes the reduction of excess contrast and noise (CNR-based), and the enhancement of subtle contrast and edges^25^. The separate frequency bands are reconstructed to an image by a mathematical algorithm called multi-scale inverse transformation. After further gradation processing the final image is displayed^25^. Usually details for the post-processing are adapted to the characteristics of the specific radiography system. For this study the default presets provided by the manufacturer were used.

### Qualitative evaluation of image quality

Three radiologists with 5 years of experience in skeletal radiography independently compared image pairs regarding the quality of the depiction of anatomical structures and overall image quality regarding the “European Guidelines on quality criteria for diagnostic radiographic images”^27^. An image pair consists of images post-processed with either MUSICA 2 or MUSICA 3 from the same raw image data. We have considered and believe that between MUSICA 3 and MUSICA 2 images are no distinguishing characteristics/image appearance/features, so that the reading has been truly blinded. Each image of a pair was displayed on a particular monitor calibrated to the DICOM greyscale standard and officially certified for diagnostic image reporting (EIZO, Hakusan, Japan) in a random order. The monitors used have a 4 k resolution and 31.5″ diagonal diameter. Two identical certified monitors of the same manufacturer and model were used. The evaluators were blinded to the post-processing technique for each image. The preset standard window width/level was identical for M2 and M3. No image manipulation was permitted. Room lighting was dimmed (20–38 lx).

The evaluator’s task was to compare the image on the left screen with the reference image on the right screen, using a 5-point scale (− 2: significantly worse, − 1: worse; 0: equal; + 1: better, + 2 significantly better), known as visual relative grading of image quality. For rating the general image quality the criteria based on the guidelines published by the European Guidelines on Quality Criteria for Diagnostic Radiographic Images, 1996^27^ were considered rather than the subjective criteria such as whether the appearance of the image is pleasing to the eye. An image of high quality is characterized by a small amount of noise, a high edge definition and a high resolution of small details. An image of poor quality on the contrary is characterized by a high amount of noise, blurry edges and a low resolution of small details. Anatomical structures to be evaluated included a joint (sacroiliac joint in pelvis, femorotibial joint space in the knee and upper ankle joint in ankles), a protruding bony landmark (major and minor trochanter in pelvis, tibial tuberosity in the knee, lateral malleolus in ankles) and the fat-muscle layers of the leg. For visual absolute grading of image quality, the evaluators state their opinion on the visibility of a certain feature without a reference image, using the scores from 1 to 5 (1 is “non-diagnostic”, 2 is “less than average”, 3 is “average”, 4 is “above average” and 5 is “excellent”). Furthermore, a quantitative assessment of IQ in terms of Signal-to-Noise-Ratio (SNR) and contrast-to-Noise-Ratio (CNR) derived from specific anatomical structures of the images was performed.

SNR was defined as:

$$SNR=\frac{{\mu }_{target}}{{\sigma }_{bg}}$$

CNR was defined as:

$$CNR= \frac{|{\mu }_{target}-{\mu }_{ref}|}{\sqrt{{\sigma }_{target}^{2}+{\sigma }_{ref}^{2}}}$$

µ_target_ and µ_ref_ denote the mean of signal in a certain region of interest (ROI) at the target structure and the reference (ref) tissue, respectively. σ_target,_ σ_ref_ and σ_bg_ represent the standard deviation of the according signal in the ROI of the target structure and reference tissue and background (bg) region, respectively. The circular ROI were manually chosen areas of 4 cm^2^. The ROI selection follows the previous study^20^.

### Statistical evaluation

All statistical analyses were carried out with “IBM SPSS Statistics 23” (IBM Corporation, Endicott, NY, USA). Significance level α was set at 0.05 with p < α.

The results of the subjective evaluation of the depiction of the anatomical structures and overall image quality were descriptively analyzed including the calculation of average, standard deviation, median, 25th and 75th percentile. The null hypothesis proves that image quality is equal in both MUSICA 2 post-processed (M2-) and MUSICA 3 post-processed (M3-) radiographs. To confirm the alternative hypothesis that image quality of M3-radiographs is higher than in M2-radiographs, a Wilcoxon Test for non-parametric, single samples on the median grades was performed with a value of significance α of 0.05. If the median grade significantly differs from grade 0 (= equal image quality) the alternative hypothesis is confirmed.

The Intraclass Correlation Coefficient (ICC) was calculated to evaluate the agreement between the evaluators. An ICC near value 1 speaks for a good agreement between the evaluators and justifies the pooling of the given grades. A poor agreement (ICC near value 0) has the consequence of separate subsequent analyses.

A Wilcoxon Test for non-parametric, paired samples was performed to analyze whether the results of each corresponding image pair are concordant. This is to analyze whether the order of display of the images or a particular monitor has an impact on the grades. All image pairs were shown twice to the evaluators, once a M2-radiograph as reference image on the right screen and once as image to be evaluated on the left screen, vice versa for M3-radiograph. If the evaluator gave a grade of + 1 (better) to the M2-radiograph on the left screen, for example, a grade of − 1 (worse) given to the M3-radiograph on the left screen would be consistent, if the order of display has no impact on the evaluation. If the evaluator gave a grade of + 1 (better) to the M2-radiograph on the left screen, but gave grade of + 1 (better) to the M3-radiograph on the left screen, too, then this evaluation indicates a preference of the left screen rather than the impact of MFP.

All grades given in the constellation M2-radiograph compared to M3-radiograph as reference were multiplied with − 1 for better comparability with grades given in the constellation M3-radiograph compared to M2-radiograph as reference. An insignificant result in the Wilcoxon Test excludes a bias caused by a particular monitor or order of display.
